# Association Between Time Preferences and Masticatory Function in Community‐Dwelling Older Adults: A Cross‐Sectional Study

**DOI:** 10.1111/joor.13984

**Published:** 2025-04-29

**Authors:** Kohei Yamaguchi, Ayane Horike, Kanako Toda, Rieko Moritoyo, Kanako Yoshimi, Kazuharu Nakagawa, Haruka Tohara

**Affiliations:** ^1^ Department of Dysphagia Rehabilitation Institute of Science Tokyo Tokyo Japan; ^2^ Department of Health Sciences Saitama Prefectural University Saitama Japan

**Keywords:** behavioural economics, cross‐sectional studies, delay discounting, mastication, nutrition assessment, procrastination

## Abstract

**Background:**

Eating is fundamental to life, encompassing not only nutritional intake but also emotional, social and communicative functions. Since the global ageing population grows, assessing eating ability‐related issues, particularly masticatory dysfunction, is of significance. Masticatory dysfunction impairs food absorption, elevates the risk of choking and contributes to malnutrition. Time preferences, such as time discounting and present bias, influence health behaviours and may be related to masticatory function; however, this relation remains unclear.

**Objective(s):**

This study aimed to investigate the association between time preference and masticatory function in community‐dwelling older adults.

**Methods:**

This cross‐sectional survey included older adults (≥ 65 years) participating in senior clubs in an urban area of Japan. Time preferences (time discounting and procrastination) were assessed using selected questions from the Global Preference Survey, while masticatory function was measured by the Gluco Sensor GS‐II. Nutritional status was evaluated by the Mini‐Nutritional Assessment‐Short Form. Linear regression analyses were conducted to assess the association between time preference and outcomes.

**Results:**

Among the participants (*N* = 65, mean age 79.1 ± 5.5 years), a significant association was found between higher time discounting tendencies and lower masticatory function (β = −19.62, *p* = 0.014). Procrastination was not associated with masticatory function or nutritional status.

**Conclusion:**

Higher tendencies towards time discounting were associated with lower masticatory function, suggesting that cumulative behavioural decisions might influence oral function. These findings provide a foundation for future research and interventions aimed to promote behavioural changes for improving functional outcomes.

## Graphical abstract

This study highlights the association between time preference and masticatory function in older adults. Individuals who more steeply discount future value tended to have lower masticatory function. These findings offer new insights into how decision‐making tendencies relate to oral function in aging populations.

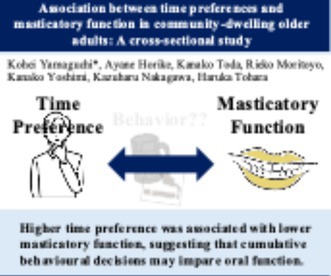

## 1 Introduction

Eating is an essential part of life. It not only serves as a means of nutritional intake but also evokes emotions, facilitates communication and supports social interaction [1, 2]. In Japan, a super‐ageing society, approximately 30% of the population is aged 65 years or older; the global ageing rate is projected to double between 2019 and 2050 [3]. In an ageing world, challenges related to eating ability are increasing. The oral cavity is part of the digestive tract that is responsible for chewing and swallowing. Mastication is the first step in the digestive process; thus, impaired masticatory function can adversely affect food absorption [4]. Decreased masticatory function not only increases the risk of choking [5] but also contributes to malnutrition. This is due to decreases in the variety of foods ingested [5] such as non‐starch polysaccharides, proteins, calcium, iron, niacin, vitamin C etc. [6], worsening nutritional status [7].

According to behavioural economics, it has been shown that when people make decisions, they make both rational and irrational judgements influenced by biases and preferences. Intertemporal decision‐making, particularly when comparing present and future choices, often leads to undervaluing future benefits in favour of immediate rewards. This tendency is known as time preference, encompassing concepts such as time discounting tendency and present bias [8]. Procrastination, driven by these factors, involves voluntarily delaying an action despite an awareness of potential negative consequences [9, 10]. It has been linked to various health‐related behaviours, such as avoiding medical examinations or smoking [11]. Human decision‐making, particularly concerning health behaviours, is characterised by uncertainty and bounded rationality, leading to irrational behaviours due to certain cognitive distortions or biases. Despite knowing the risks to their health, individuals may still smoke, drink excessively and become obese [12, 13].

The accumulation of decisions on health behaviours may lead to functional decline and, eventually, more serious disabilities. However, the association between time preference and physical functioning remains unclear. In particular, older people may experience heightened consequences from accumulated health behaviours shaped by time preferences [14]. Despite this, there is a significant lack of knowledge about the time preferences of older people. Understanding the association between time preference and masticatory function would enable early identification of behavioural biases and prompt intervention to prevent frailty in older people, as the oral cavity is the gateway to systemic frailty, such as malnutrition [15]. Therefore, this study aimed to clarify the association between time preference and masticatory function in older adults. Our hypothesis was that older people with strong tendencies for procrastination would also have poor masticatory function and an increased risk of malnutrition.

## 2 Methods

### 2.1 Research Design and Settings

The cross‐sectional survey included community‐dwelling older adults. It was conducted at a senior club organisation in a central urban district that balanced historical neighbourhoods with modern commercial areas. The broader region, situated in one of Japan's major metropolitan areas with a population of several million, serves as a key port and business hub. The survey was conducted between November 2022 and February 2023. No prior sample size calculation was performed specifically in this study, as the dataset was originally collected to investigate the association between dental health behaviours and preferences.

### 2.2 Participants

The inclusion criteria encompassed community‐dwelling older adults aged 65 years or older who participated in a senior club and attended measurement sessions conducted by the researchers. Individuals with significant difficulty in following instructions, acute illness or significant malnutrition were excluded from the study.

### 2.3 Time and Risk Preferences

The study assessed preferences, including time discounting rate, procrastination and risk preferences. Time preferences were evaluated using questions selected from the Global Preference Survey (GPS) [16]. Procrastination was measured by the question: ‘Generally speaking, how often do you put off something you should do, even though you know it would be better to do it now’? Responses were rated on a 10‐point Likert scale, with 10 representing ‘very often’ and 0 representing ‘not at all’. Similarly, time discounting tendencies were evaluated by the question: ‘Generally speaking, how willing are you to let go of what currently benefits you for a greater future benefit?’. Responses ranged between 10 (‘not willing at all’) and 0 (‘very willing’). Risk preference was assessed by the question, ‘Generally speaking, how willing are you to take risks?’ and scored on the same scale, with 10 representing ‘not willing at all’ and 0 representing ‘very willing’. The participants were asked to select the option that best applied to each question.

### 2.4 Primary and Secondary Outcomes

The primary outcome, masticatory function, was assessed by a Gluco Sensor GS‐II (GC, Tokyo, Japan). The participants chewed a glucose‐containing gummy for 20 s, gargled with 10 mL of water and the gummy and water mixture was passed through a filter mesh. The amount of glucose eluted from the solution was measured [17]. A series of measurements were performed by dentists, dental hygienists and others with sufficient experience.

The secondary outcome, nutritional status was assessed by the Mini‐Nutritional Assessment‐Short Form (MNA‐SF) [18], a simple questionnaire comprising six questions with a maximum score of 14. A score of 12–14 indicated ‘good nutritional status’, a score of 8–11 indicated ‘at risk of malnutrition’ and a score of 7 or less indicated malnutrition.

### 2.5 Covariates

In addition to basic information, such as age and sex, the number of remaining teeth was recorded. An experienced dental hygienist confirmed the number of remaining teeth. Financial sense, as a proxy for general financial status, was assessed by the question: ‘How much would one be willing to pay for a meal alone on a weekday?’ To answer this question, respondents had to choose one of nine options ranging between 499 yen or less and 15 000 yen or more.

### 2.6 Statistics

The mean value ± standard deviation of each item is described, including basic information such as the participant's time preferences (procrastination and time discounting tendency), risk preference, financial sense, age, sex and number of remaining teeth. Spearman correlation coefficients were calculated to assess the correlation between time preferences (procrastination and time discounting tendency), risk preferences, masticatory function and nutritional status.

Both univariable and multivariable linear regression analyses were conducted to investigate the association between time preference and the primary and secondary outcomes. The objective variables were masticatory function and nutritional status, whereas the explanatory variables included procrastination, time discounting tendency, risk preference, financial sense, age, sex and number of remaining teeth. Sex was coded as a binary variable, where 0 represented male and 1 represented female; the other variables were treated as continuous variables. Time and risk preferences were standardised and used in all analyses. The forced entry method was used; the analysis was performed using SPSS version 25 (IBM Inc., Tokyo, Japan).

### 2.7 Ethical Considerations

This study was approved by the Institute of Science Tokyo Ethics Review Committee (D2019‐082). The participants were fully informed about the study orally or in writing and provided written consent.

## 3 Results

A total of 72 individuals participated in the study. After excluding five participants who did not complete the questionnaire, one who declined to participate and another with missing data, the final sample included 65 participants.

The participants had a mean age of 79.1 ± 5.5 years. The mean values of the participants' time preferences were 3.6 ± 2.4 for procrastination and 5.2 ± 3.1 for time discounting tendency (Table 1). Procrastination was not correlated with masticatory function or the MNA‐SF. Time discounting tendency was correlated with masticatory function (*r* = −0.262, *p* < 0.05), without correlation with MNA‐SF (Table 2).

**TABLE 1:** Descriptive characteristics of participants (*N* = 65).

	Total	Range
Procrastination, points	3.6 ± 2.4	0–10
Time discounting tendency, points	5.2 ± 3.1	0–10
Risk preference, points	6.6 ± 2.4	0–10
Age, years	79.1 ± 5.5	66–90
Sex, *n* (%)	Men, 65 (40%)	
Economic indicators, points	4.4 ± 1.5	2–9
Number of remaining teeth	19.8 ± 8.8	0–28
Masticatory function, mg/dL	196.8 ± 69.9	55–335
MNA‐SF, points	12.2 ± 2.3	1–14

Abbreviation: MNA‐SF, Mini Nutritional Assessment‐Short Form.

**TABLE 2:** Correlation coefficient between preferences, economic indicators and dependent variables (*N* = 65).

	Masticatory function	MNA‐SF
Procrastination	−0.058	−0.201
Time discounting tendency	−0.262^a^	0.093
Risk preference	−0.060	0.118
Economic indicators	0.340^b^	0.154

*Note:* A high score on procrastination and time discounting tendency indicates a greater tendency towards procrastination and time discounting. A low score on risk preference indicates risk‐seeking behaviour.

Abbreviation: MNA‐SF, Mini Nutritional Assessment‐Short Form.

^a^ The correlation coefficient is significant at the 5% level.

^b^ The correlation coefficient is significant at the 1% level.

Univariable and multivariable linear regression analyses were performed to assess the association between time preference and masticatory function. Regarding the univariable analysis, no significant association was found between time discounting tendency and masticatory function. However, sex (Β [95% confidence interval {CI}] = 38.41 [4.08–72.74], *p* = 0.03) and the number of teeth (Β [95% CI] = −4.48 [−6.13 to −2.82], *p* < 0.001) were significantly associated (Table 3). Considering the multivariable linear regression model with masticatory function as the dependent variable, time discounting tendency (Β [95% CI] = −19.62 [−35.20 to −4.05], *p* = 0.014), financial sense (Β [95% CI] = 11.49 [1.74–21.24], *p* = 0.022) and the number of remaining teeth (Β [95% CI] = 4.33 [2.52–6.13], *p* < 0.001) were independently related factors (Table 3). The standard partial regression coefficient for time discounting tendency was 0.42. When the MNA‐SF was used as the dependent variable, the univariable linear regression analysis yielded no significant results. Furthermore, no significant models were identified using the multivariable linear regression analysis (Table 4), as indicated by the *p* value of 0.08 from the analysis of variance.

**TABLE 3:** Association between time preferences and masticatory function by univariable and multivariable regression analysis (*N* = 65).

	Univariable model					Multivariable model				
	*B*	95% CI		*p*	Adjusted *R* ^2^	*B*	95% CI		*p*	Adjusted *R* ^2^
Age	−0.102	−3.296	3.092	0.949	−0.016	1.890	−0.613	4.394	0.136	0.418
Sex	38.410	4.078	72.743	0.029	0.059	2.159	−27.867	32.185	0.886	
Procrastination	−7.439	−24.949	10.071	0.399	−0.004	−12.947	−27.257	1.363	0.075	
Time discounting tendency	−16.102	−33.238	1.035	0.065	0.038	−19.624	−35.200	−4.049	0.014	
Risk preference	−2.838	−20.433	14.758	0.748	−0.014	−0.586	−16.520	15.348	0.942	
Economic indicators	16.507	5.565	27.449	0.004	0.112	11.488	1.739	21.238	0.022	
Number of remaining teeth	4.478	2.824	6.131	< 0.001	0.307	4.328	2.524	6.133	< 0.001	

*Note:* A high score on procrastination, time discounting indicates higher level of procrastination tendency and time discounting tendency. A low score on risk preference indicates risk‐seeking attitudes.

Abbreviations: B, unstandardized coefficient; CI, confidence interval; *p*, *p*‐value.

**TABLE 4:** Association time preferences and MNA‐SF by univariable and multivariable regression analysis (*N* = 65).

	Univariable model					Multivariable model				
	*B*	95% CI		*p*	Adjusted *R* ^2^	*B*	95% CI		*p*	Adjusted *R* ^2^
Age	−0.101	−0.203	0.001	0.053	0.043	−0.092	−0.195	0.011	0.078	0.093
Sex	0.154	−1.021	1.329	0.794	−0.015	−0.412	−1.648	0.824	0.507	
Procrastination	−0.556	−1.119	0.008	0.053	0.043	−0.673	−1.262	−0.084	0.026	
Time discounting tendency	0.210	−0.368	0.788	0.470	−0.007	0.165	−0.476	0.806	0.608	
Risk preference	0.162	−0.417	0.741	0.577	−0.011	−0.02	−0.675	0.636	0.953	
Economic indicators	0.265	−0.115	0.645	0.169	0.014	0.359	−0.042	0.761	0.078	
Number of remaining teeth	0.056	−0.009	0.120	0.089	0.030	0.030	−0.044	0.105	0.416	

*Note:* A high score on procrastination and time discounting indicates a higher level of procrastination tendency and time discounting tendency. A low score on risk preference indicates risk‐seeking attitudes.

Abbreviations: B, unstandardized coefficient; CI, confidence interval; MNA‐SF, Mini Nutritional Assessment‐Short Form; *p*, *p*‐value.

## 4 Discussion

In this study, higher time discounting tendency was an independent factor associated with lower masticatory function; however, no association was found with procrastination. No statistically significant results were obtained regarding the MNA‐SF scores.

Time discounting tendency emerged as an independent factor related to masticatory function; the participants with higher time discounting tendencies exhibited lower masticatory function. However, no association was found with procrastination. Previous research shows that people with high time discounting tendencies undervalue future rewards, making it challenging for them to engage in behaviours that benefit their future health [11, 12, 13]. It has been reported that masticatory function is associated with tooth loss and that procrastination in childhood is associated with the number of teeth in old age [14]. The masticatory function is currently related to the number of teeth and tongue, lip and whole‐body function [19, 20]. Additionally, individuals with high time discounting tendencies often struggle to maintain regular exercise habits [21], which can increase the risk of oral and systemic frailty [22, 23]. The association between procrastination and time discounting tendencies has not yet been fully clarified; although time discounting rates are time consistent, procrastination is not [20, 24]. Individuals with high time discounting tendencies may struggle to take action for uncertain future health outcomes due to present bias, which prevents them from taking action even if they are interested in health behaviour. Consequently, a lack of engagement in health behaviours can accumulate over time, ultimately impacting functional outcomes. A negative trend was found between the MNA‐SF scores and procrastination, suggesting that higher procrastination may be linked with an increased risk of malnutrition; however, this association was not statistically significant. Since masticatory function is associated with malnutrition [25], it is plausible that time preference may exhibit similar associations.

The mechanisms underlying the association between time preference and masticatory function can be explained by two potential pathways. First, time preferences may influence masticatory function through health‐related behaviours such as dental visits and oral hygiene management [26]. Second, masticatory function itself may activate brain regions involved in decision‐making, such as the prefrontal cortex [27, 28, 29]. Based on these two assumptions, this association was hypothesised to be bidirectional. Individuals with low time discounting tendencies are more likely to engage in health‐promoting behaviours, such as exercise and outdoor activities [30]. A previous study has shown that systemic exercise is strongly associated with masticatory function in older adults [31]. On the other hand, neuroeconomics studies, using functional magnetic resonance imaging, demonstrate that future‐oriented decision‐making primarily activates the dorsolateral prefrontal cortex [32], which also benefits from increased blood flow during chewing [33]. This positively influences cognitive function [34]. These findings suggest that brain activity may mediate the association between time preference and masticatory function.

Using the GPS across 76 countries shows that time discounting decreases with age, peaking at middle age, with some sex differences that vary across cultural contexts, while women and older individuals are more risk‐averse than men and younger ones, respectively [16]. This study, including a notably older population (mean age: 79.1 ± 5.5 years), found low procrastination, no strong bias in time discounting and a tendency towards risk aversion, aligning with GPS trends and previous studies [35]. In today's ageing society, future research that explores the characteristics of older adults' preferences and their relationship with health status is expected to become increasingly important.

This study has some limitations. Its small sample size and lack of cognitive function assessment might limit the ability to establish a causal association between time preference and masticatory function. Further studies with larger sample sizes, longitudinal designs and cognitive assessments are needed for confirming these findings. Also, nutritional status was evaluated subjectively using questionnaires. More detailed nutritional evaluations, such as the Global Leadership Initiative on Malnutrition criteria [36], were not performed. However, as the participants were community‐dwelling older adults, none appeared to have severe malnutrition; their mean MNA‐SF score was 12.2 ± 2.3, indicating a well‐nourished population. Despite these limitations, this study is the first to demonstrate the association between time preferences and masticatory function, offering foundational knowledge for building behavioural change models with higher predictive accuracy.

In addition, this study provides useful insights for clinicians and policymakers. The association between time and masticatory function suggests that specific preferences can serve as useful indicators of masticatory function. Since a decline in oral function and specific preferences are likely gateways for systemic functional decline [15], early detection of such issues is critical. Screening individuals for time preferences could allow the identification of general trends without the need for extensive functional tests, potentially enabling not only healthcare professionals but also nonmedical personnel or even community members to recognise potential risks in others, fostering mutual awareness and preventive action. The findings of this study might be particularly valuable for population‐level health promotion strategies such as nudging [37].

## 5 Conclusion

This study demonstrated that in community‐dwelling older adults, time preference was independently associated with masticatory function. Although the association between time preference and nutritional status was not statistically significant, a negative trend was observed, suggesting a potential link. These findings provided important information on the association between preferences and functional outcomes, which might reflect the cumulative impact of behaviours over time. Furthermore, they underscored the potential utility of approaches targeting behavioural changes in improving functional and health outcomes.

## Author Contributions

**Kohei Yamaguchi:** conceptualisation, methodology, investigation, original draft preparation, writing, reviewing and editing. **Ayane Horike:** methodology, investigation, data curation and reviewing. **Kanako Toda:** data curation, investigation, validation and editing. **Rieko Moritoyo:** methodology, data curation, investigation and validation. **Kanako Yoshimi:** methodology, reviewing and validation. **Kazuharu Nakagawa:** methodology, reviewing and editing. **Haruka Tohara:** reviewing, editing and supervision.

## Conflicts of Interest

The authors declare no conflicts of interest.

## Peer Review

The peer review history for this article is available at https://www.webofscience.com/api/gateway/wos/peer‐review/10.1111/joor.13984.

## Data Availability

The data that support the findings of this study are available from the corresponding author upon reasonable request.
